# Cognitive bias modification of interpretation in children with social anxiety disorder

**DOI:** 10.1016/j.janxdis.2016.10.012

**Published:** 2017-01

**Authors:** Faith Orchard, Adela Apetroaia, Kiri Clarke, Cathy Creswell

**Affiliations:** a School of Psychology and Clinical Language Sciences, University of Reading, Whiteknights Road, Reading, RG6 6AL, United Kingdom; b Newham College University Centre, London, E15 4HT, United Kingdom; c Division of Psychology and Language Sciences, University College London, London, WC1E 6BT, United Kingdom

**Keywords:** Social anxiety disorder, Interpretation, Cognitive bias modification, Child

## Abstract

Negative (or a lack of positive) interpretation of ambiguous social situations has been hypothesised to maintain social anxiety disorder in children, yet there is currently limited evidence to support this. Cognitive Bias Modification of Interpretation (CBM-I) provides a means to explore the causal influence of interpretation bias on social anxiety disorder, and has been associated with a reduction in social anxiety symptoms in adults. Seven to twelve year old children with a diagnosis of social anxiety disorder completed CBM-I training, adapted from materials designed for socially anxious children in the community, or no training. Effects on interpretation bias and social anxiety were assessed. The adapted CBM-I training was not associated with significant changes in benign or negative interpretation. Unsurprisingly given the lack of successful interpretation training, there were no significant changes in child or parent reported social anxiety symptoms, clinician-rated severity or diagnoses and change in interpretation was not significantly associated with change in social anxiety. These findings contrast with some studies with community populations although it is possible that more intensive CBM-I training is required to fully test this hypothesis among clinical groups.

## Highlights

• Children with social anxiety disorder were assigned to CBM-I training or no training.
• We measured the effects on interpretation bias and social anxiety.
• CBM-I was not associated with greater change in interpretations than no training.
• Social anxiety symptoms and diagnoses were not influenced by CBM-I training.
• More intensive CBM-I training may be required.

## 1 Introduction

Social anxiety disorder is common in children (Beesdo et al., 2007), causes significant functional impairment (Erath, Flanagan, & Bierman, 2007; Van Ameringen, Mancini, & Farvolden, 2003) and is associated with long term risk of adult social anxiety as well as other mental health difficulties (Pine, Cohen, Gurley, Brook, & Ma, 1998; Zimmermann et al., 2003). Social information is often ambiguous, yet adults without social anxiety disorder often interpret this information in a positive manner. This helpful positive bias is lacking in adults with social anxiety disorder (Hirsch and Mathews, 2000, Stopa and Clark, 2000) and it is hypothesised that a lack of positive interpretation bias may play a fundamental role in the maintenance of social anxiety disorder (Hirsch & Clark, 2004).

Psychological treatments for social anxiety disorder among children typically include methods which aim to change interpretation bias (e.g. NICE, 2013). However it is far from clear that interpretation biases have a maintaining role in childhood social anxiety disorder as studies to date have typically examined cross-sectional associations, and where these have been conducted they have not provided consistent evidence for specific associations between social interpretation biases and social anxiety disorder (e.g. Muris, Kindt et al., 2000). For example, Creswell, Murray & Cooper (2013) failed to find differences in either the frequency of threat interpretation in responses to ambiguous social scenarios, or in expectations of how a social challenge would turn out in children (7–12 years) with social anxiety disorder, other anxiety disorders and non-anxious children. However, although social anxiety disorder in adults is characterised by a lack of positive bias (e.g. Hirsch & Mathews, 2000; Garner, Mogg & Bradley, 2006), studies with children have typically failed to distinguish between increased positive and reduced negative interpretations, instead tending to treat these as on a continuum (e.g. Creswell et al., 2013). Furthermore, there has been limited examination of the prospective relationship between cognitions and social anxiety in children (Muris, Huijding, Mayer, Remmerswaal, & Vreden, 2009).

One method that has the potential to determine causal influences of interpretation on social anxiety symptoms is Cognitive Bias Modification of Interpretation (CBM-I) which involves training participants to interpret ambiguous social stimuli in a more positive and/or less negative fashion. CBM-I has been associated with reduced social anxiety symptoms in both community and clinical adult populations (e.g. Murphy, Hirsch, Mathews, Smith, & Clark, 2007; Beard & Amir, 2008), and there is evidence that change in benign interpretation mediates the effect of training on social anxiety (Beard & Amir, 2008).

Recent applications of CBM-I with children have led to mixed results. On the basis of a meta-analysis of CBM (of attention and interpretation) interventions for mental health problems in children, Cristea, Mogoase, David and Cuijpers (2015) concluded that while CBM appeared to bring about significant changes in interpretation biases, these shifts did not translate to changes in child symptoms of anxiety, depression or general mental health (consistent with recent studies of Attention Bias Modification in the context of social anxiety; Carleton et al., 2015; Heeren, Mogoaşe, McNally, Schmitz & Philippot, 2015; Yao, Yu, Qian & Li, 2015). However, interpretation measures were collapsed to include both controlled in-lab experiments and real-life, ecologically valid measures, leaving the extent to which interpretation bias changed somewhat unclear. Where studies have focused on CBM-I and social anxiety specifically, findings have been mixed. One study reported a reduction in trait social anxiety among twenty two 10–11 year old children from a community population who scored highly on trait social anxiety, after 3 sessions of CBM-I with 45 trials (Vassilopoulos, Banerjee & Prantzalou, 2009). Yet, another study found no training effects on trait social anxiety among 77 10–13 year old children, despite using similar methods (Vassilopoulos, Moberly, & Zisimatou, 2013). In both studies, interpretation bias training was associated with reduced negative interpretation (Vassilopoulos et al., 2009, Vassilopoulos et al., 2013) and in Vassilopoulos et al. (2013) training was also associated with more benign interpretation of ambiguous social scenarios. One possible reason why CBM-I may have failed to translate to a change in social anxiety symptoms in Vassilopoulos et al. (2013) is that participants were an unselected sample who did not have elevated levels of social anxiety at the outset. Indeed, other studies with unselected populations have managed to successfully train interpretation biases but have failed to find an effect on general anxiety symptoms in children (Lester, Field, & Muris, 2011; Salemink & Wiers, 2011; though see Hirsch, Mathews & Clark, 2007, for contrasting evidence in adults); and it has been suggested that symptoms may be more likely to change following CBM-I for highly anxious child populations (e.g. Cristea et al., 2015; Muris, Huijding, Mayer, & Hameetman). No studies to date have applied CBM-I with children who meet diagnostic criteria for social anxiety disorder, however, if successful in reducing social anxiety symptoms, this provides both valuable insights in to the relationship between interpretation and social anxiety and may lead to low-intensity treatment options (e.g. Beard, Weisberg, & Amir, 2011).

We aimed to address whether CBM-I is associated with more benign interpretation and less negative interpretation of ambiguous social scenarios, a reduction in social anxiety symptoms and severity, and whether change in social anxiety was mediated by changes in interpretation. Some particular methodological features of the current study should be noted. We aimed to use an established method of CBM-I for social anxiety which had previously been used with high socially anxious children recruited from the community (Vassilopoulos et al., 2009). However, given previous CBM-I training programmes for social anxiety in children have relied heavily on children’s reading abilities and have not used highly systematised procedures (instead presenting training materials on cards which are read, circled and then turned over by participants to reveal ‘correct’ interpretations and therefore varying in timing of presentation, manner in which materials are read etc, e.g. Vassilopoulos et al., 2009, Vassilopoulos et al., 2013) we adapted these previously used training materials for administration using experimental software with accompanying audio-materials. Furthermore, we asked participants to imagine themselves in the described scenarios since this has been shown to augment CBM-I in adults with depression (Holmes, Lang, & Shah, 2009).

The study hypotheses were as follows:

1. Children who receive the CBM-I training will have more benign interpretation and less negative interpretation post-training compared to children who do not receive CBM-I training.
2. Children who receive the CBM-I training will have lower scores on child, parent and clinician ratings of social anxiety post-training, compared to children who do not receive CBM-I training.
3. The association between group (‘training’, ‘no training’) and change in social anxiety will be mediated by the change in benign and negative interpretation biases, i.e., confirming a causal relationship between interpretation biases and anxiety.

## 2 Materials and methods

On the basis of the large effect found in Vassilopoulos et al. (2009), 30 participants were required to conduct repeated measures analyses of variance investigating within-between interactions (effect size *F* *=* 0.35; power 95%; correlation among repeated measures, 0.48, as reported below). However, as an effect size has yet to be obtained with a clinical group, we powered this study for a medium effect size (*F* = 0.25), and so recruited n = 56 participants in order to provide at least 90% power to detect this more conservative effect.

### 2.1 Participants

**Fig. 1:**
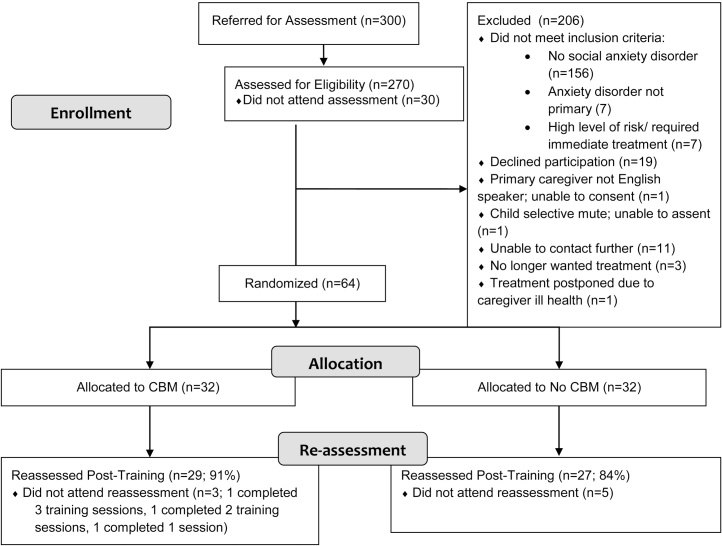
Participant flow, randomisation, withdrawals and exclusions at each stage of the study. Note. CBM: Children who took part in cognitive bias modification training; No CBM: children who did not take part in cognitive bias modification training.

All participating children had been referred to the Berkshire Child Anxiety Clinic at the University of Reading by health or education professionals for assessment and treatment of an anxiety disorder. Children were invited to take part if they met the following inclusion criteria: (i) a primary anxiety disorder and a diagnosis of social anxiety disorder, (ii) aged 7–12 years, (iii) fluent English speakers, (iv) absence of learning difficulties (including autistic spectrum disorder), (v) absence of severe condition or risk that requires immediate treatment. A flow diagram showing recruitment and retention is shown in Fig. 1. Fifty six clinically anxious children and their primary caregivers (all mothers) gave informed consent and took part in all stages of the study. Participants were randomized, using a random number generator, to either receive training (CBM) or not to receive training (NO CBM). The groups were well balanced on child age, gender, ethnicity, socioeconomic status, and symptoms of anxiety and low mood (see Table 1). No significant difference was found between groups for child anxiety disorder by comparing primary diagnosis (χ^2^(6) = 4.68, *p* = 0.59) and frequency of social anxiety as the primary disorder (χ^2^(1) = 0.25, *p* = 0.62). Groups did not differ on the presence of externalizing disorders (χ^2^(1) = 2.70, *p* = 0.10) or mood disorders (χ^2^(1) = 1.46, *p* = 0.23). (See Table 2 for frequencies of primary anxiety diagnoses and overall diagnoses). There was also no difference between the CBM group (*M* = 5.48, *SD* = 0.63) and the NO CBM group (*M* = 5.37, *SD* = 0.74) on ADIS-C/P clinical severity ratings of the primary diagnosis (*t*[54] = −0.61, *p* = 0.54).

### 2.2 Measures

#### 2.2.1 Anxiety disorders interview schedule for DSM IV for children–child and parent versions (ADIS-C/P; Silverman & Albano, 1996)

Children were assigned diagnoses on the basis of the ADIS-C/P, a structured diagnostic interview with well-established psychometric properties (Silverman, Saavedra, & Pina, 2001). The interview covers anxiety disorders, mood disorders (depression and dysthymia) and behavioural disorders (conduct disorder, oppositional defiant disorder and attention deficit hyperactivity disorder). Where children met symptom criteria for a diagnosis (based on either child or parent report) they were assigned a clinical severity rating (CSR) ranging from 0 (*complete absence of psychopathology*) to 8 (*severe psychopathology*). As is conventional, only those children who met symptom criteria with a CSR of 4 or more (*moderate psychopathology*) were considered to meet diagnostic criteria. For the ADIS-C/P, as is standard, overall diagnoses and CSRs were assigned if the child met diagnostic criteria on the basis of either child or parent report, and the higher CSR of the two was taken. Assessors (psychology graduates) were trained on the standard administration and scoring of the ADIS-C/P through verbal instruction, listening to assessment audio-recordings and participating in diagnostic consensus discussions. The first 20 interviews conducted were then discussed with a consensus team, led by an experienced diagnostician (Consultant Clinical Psychologist). The assessor assigned diagnoses and CSRs prior to the consensus meeting and the consensus team independently allocated diagnoses and CSRs after the discussion. It is worth noting that the consensus discussions were limited by a reliance on how the team interpreted the interviewer’s description, however audio recordings of ADIS-C/P interviews were available and were used to inform discussions/check decisions at times. Following the administration of 20 interviews, interrater reliability for each assessor was checked, and if assessors achieved reliability of at least 0.85, they were then required to discuss just one in six interviews with the consensus team (ongoing checks were conducted to prevent interrater drift). All assessors were reliable after 20 interviews. Overall reliability (on the first 20 and the subsequent interviews that were discussed) was conducted for the assessment team and was found to be excellent (Diagnosis α = 0.98 (child report), α = 1.00 (mother report); CSR α = 0.91 (child report), α = 0.97 (mother report)).^1^

#### 2.2.2 Spence Children’s Anxiety Scale (SCAS-C/P; Nauta et al., 2004, Spence, 1998)

The SCAS-c/p requires children/parents to rate how often they/their child experience/s each of 38 anxiety symptoms (presented alongside six positive filler items in the child report version) on a 4 point scale from 0 (*never*) to 3 (*always*). The social phobia scale of the SCAS-C/P was used as an indicator of self- and parent- reported social anxiety symptoms. The social phobia scale of the SCAS has been found to correlate highly with the Social Anxiety Scale for Children (SASC-R; Muris, Merckelbach, & Damsma, 2000). Internal consistency was acceptable to good (SCAS-c α = 0.84; SCAS-p α = 0.84; SCAS-SP-c α = 0.69; SCAS-SP-p α = 0.80).

#### 2.2.3 Short mood and feelings questionnaire (SMFQ-C/P; Angold et al., 1995)

In order to assess the severity of common comorbid symptoms, and identify potential group differences, the SMFQ-c/p was administered to assess child and parent reported low mood. The SMFQ is a brief, 13 item measure which requires children/parents to report how often in the past two weeks the child has experienced a number of depressive symptoms on a 3 point scale from 0 (*not true*) to 2 (*certainly true*). Internal consistency was good (SMFQ-c α = 0.80; SMFQ-p α = 0.87).

#### 2.2.4 Ambiguous scenarios interview for children (Vassilopoulos et al., 2009)

The ambiguous scenarios interview was used as a measure of both negative and benign interpretation. Interviews were conducted by a graduate research assistant and consisted of 16 ambiguous social scenarios which reflect events that commonly occur and are relevant for participants of this age, such as inviting classmates to your birthday party, approaching a group of peers, or going to a classmate’s home to play together. Each scenario is followed by two thoughts which might occur to children in these situations reflecting a negative (for example, ‘[classmates] don’t want to come [to party] because they don’t like me’), or a benign judgement (‘they don’t know yet if they can come or not’). Children rated how likely they would be to endorse each explanation on a 5 point Likert scale. The first set of scenarios was presented at intake, the other set at the end of the training phase. Total ratings were calculated across the scenarios for each participant. The minimum score for benign or negative judgments was 8, the maximum was 40. The internal validity of the ASI overall was acceptable (benign α = 0.76; negative α = 0.77).

#### 2.2.5 CBM-I training (adapted from Vassilopoulos et al., 2009)

The CBM-I training materials consisted of a total of 45 social scenarios presented during three sessions of 15 scenarios each, using translations of the materials developed by Vassilopoulos et al. (2009). After being shown each ambiguous scenario participants were asked to select one of two alternative endings, a threatening and a non-threatening one, in counterbalanced order e.g. ‘You enter the classroom and say hello to your schoolmate. However he/she doesn’t say anything. Why do you think this happens?’ (i) She has something else on her mind and she did not hear me or (ii) She doesn’t like me anymore. Before selecting a response, participants were encouraged to reflect on the ‘correct’ interpretation. If the participants selected the non-threatening ending, they received the message ‘This is correct’, visually and aurally. If they selected the threatening interpretations, participants would see and hear the message ‘This is the correct answer:’ followed by the non-threatening ending. In both situations, participants were prompted by an audio message to think about how the non-threatening ending might explain the situation. A flow diagram representing the stages of the training procedure is shown in Fig. 1.

The procedure was adapted from Vassilopoulos et al. (2009) method in four ways. Firstly, materials were presented using E-Prime Version 2.0 rather than on paper cards. To reduce the burden of reading, participants listened to each scenario read by a female actor in a friendly, neutral voice as scenarios were presented on the computer screen, in text, at the same time. Secondly, in order for children to understand the rationale of the study they were told that they would take part in a programme that would teach them some new ways of thinking in relation to their social worries. They were introduced to the connection between thoughts and emotions before the training programme began using two hand-outs from the ‘Cool Kids’ anxiety treatment programme (Lyneham, Abbott, Wignall & Rapee, 2003). A research assistant worked through the hand-outs with the child and encouraged them to identify the thoughts and feelings in the images. In the first one, ‘The way I think and feel’, children were given examples of four situations (‘what happened?’) followed by a thought (‘what was I thinking?’) and an emotion (‘what was I feeling?’). Two of these situations were followed by a threatening interpretation, two by a non-threatening interpretation and the corresponding emotions. So as to not interfere with the training, the situations presented were non-social (for example, situation: ‘a big dog comes near me’; thought: – ‘the dog wants to play with me’; emotion –‘happy to play with the dog’). The second hand-out, ‘How I feel depends on what I think’, further explored the link between thoughts and emotions and children were encouraged to fill out examples of different thoughts and their associated emotions. As before, the situations presented were non-social. Thirdly, because accompanying imagery has been found to enhance the effects of CBM-I in studies with adult populations (Holmes et al., 2009), participants were given specific instructions to explain what an image was and to help them create a visual image of the scenarios. They were encouraged to practice imagining being at the beach, and seeing their favourite food, by concentrating on what they could see, hear, smell, feel and taste. They were then instructed to imagine that the training situation described is happening to them, even if they find it unlikely (following Lothmann, Holmes, Chan & Lau, 2011). Finally, at the end of each training item an inference based on the non-threatening interpretation is presented and the child was asked to identify it as true or false. As in CBM with adult populations (Hayes, Hirsch, Krebs & Mathews, 2010), this was intended to reinforce the non-threatening interpretation and encourage active engagement in the generation of meaning. As we only presented positive interpretations at this stage, the ‘correct’ answer was always ‘True’so the position of the ‘True’ and ‘False’ keys were varied to engage the child in thinking about the response. In keeping with some previous studies that have successfully trained altered interpretations, children did not receive feedback on these responses (e.g. Beard and Amir, 2008, Hayes et al., 2010; Micco, Henin & Hirshfeld-Becker, 2014).

### 2.3 Ethical considerations

This study was reviewed by the Local Research Ethics Committee on behalf of the National Health Service and the University of Reading Research Ethics Committee. Parents and children were both provided with written and verbal information about the study. In order to participate in the study written parental consent and child assent were both required.

### 2.4 Procedure

Children and their parents completed diagnostic interviews and symptom questionnaires as part of their routine clinical evaluation. All participants received a visit at home where they signed consent forms and completed the first set of the Ambiguous Scenarios Interview (Pre-Training) questions. Participants allocated to the CBM group were told that they would receive an experimental treatment which would teach them new ways of thinking. These participants attended a further three visits at the University to complete the CBM-I training. Each training session lasted approximately 30 min, with the first session lasting 45 min in order to also complete the hand-outs first. The sessions were spaced as evenly as possible within two weeks. Children in the NO CBM group were assigned to the waitlist condition and were not required to attend any visits until the re-assessment. All families were informed that they were on a waiting list to receive treatment as usual immediately following the CBM study, and no families reported having started any additional treatment during this time.

Reassessments were scheduled for 4 weeks following the initial home visit. However, the reassessment was completed a mean of 6.11 weeks (*SD* = 2.84) weeks after group allocation, and 1.10 weeks (*SD* = 0.34) weeks after the final training session for the CBM group. This was due to rearrangements of sessions made by families. The time from allocation to reassessment did not differ between groups (*t*(54) = 0.10, *p* = 0.92). At this reassessment all participants completed (i) the second set of the Ambiguous Scenarios Interview (Post-Training) questions, (ii) the SCAS-c/p, and (iii) the Social Phobia section of the ADIS-C/P. All post-training assessors were blind to participant group.

## 3 Results

### 3.1 Preliminary analyses

Continuous data were screened in relation to the assumptions of parametric tests (Tabachnick & Fidell, 2007). Where assumptions were violated, confirmatory analyses were conducted by running analyses with 1000 bootstrap samples or non-parametric alternatives. The majority of results were consistent, suggesting that the original analyses were robust to the violations of assumptions, so results based on the original (non-bootstrapped) analyses are presented for simplicity.

### 3.2 Change in interpretation bias

To examine hypothesis one, two mixed design analyses of variance (ANOVA) were conducted with group (CBM vs. NOCBM) as the independent variable and measures of interpretation bias as the repeated dependent variables (see Table 3). This approach was taken rather than conducting a single multivariate analysis of variance due to concerns regarding collinearity given the high correlation between benign and negative interpretation scores at time 2 (*r* = −0.51, *p* < 0.001) (Field, 2009).

Significant, large main effects of time were found for both negative (*V* = 0.12, *F*(1,53) = 7.30, *p* = 0.01; *d* = 0.72) and benign interpretation (*V* = 0.50, *F*(1,53) = 52.29, *p* < 0.001; *d* = 1.93), reflecting the fact that participants had less negative and more benign interpretation post- compared to pre-training, regardless of group. The group x time interaction effect approached significance, reflecting a trend towards a greater increase in benign interpretation among the CBM group with a medium effect size (*V* = 0.07, *F*(1,53) = 3.84, *p* = 0.055; *d* = 0.52). There was not a significant interaction between group and time for negative interpretation where the effect was small (*V* = 0.01, *F*(1,53) = 0.32, *p* = 0.58; *d* = 0.15).

### 3.3 Change in social anxiety symptoms, severity and diagnoses

To examine hypothesis two, three mixed design analyses of variance (ANOVA) were conducted with group (CBM vs. NOCBM) as the independent variable, and measures of self- and parent-reported social anxiety symptoms and clinical severity ratings of diagnoses as repeated dependent variables (see Table 3).

There was a main effect of time on child-reported social phobia symptoms on the SCAS (*V* = 0.09, *F*(1,54) = 5.09, *p* = 0.03; *d* = 0.60) reflecting a decrease in symptoms from pre- to post-training regardless of group. A significant effect was not found for parent-reported symptoms (*V* = 0.01, *F*(1,54) = 0.77, *p* = 0.39; *d* = 0.23). There was not a significant time x group interaction for either parent (*V* = 0.01, *F*[1,54] = 0.32, *p* = 0.58; *d* = 0.15) or child-reported social phobia symptoms (*V* = 0.01, *F*(1,54) = 0.28, *p* = 0.60; *d* = 0.14).

There was not a significant main effect of time on social phobia clinical severity rating (*V* = 0.01, *F*(1,54) = 0.35, *p* = 0.56; *d* = 0.16), nor a significant interaction between time and group for clinical severity rating (*V* = 0.06, *F*(1,54) = 3.27, *p* = 0.08; *d* = 0.48) and notably the pattern of results was in the opposite direction to that predicted (see Table 3).

All participants in the CBM group maintained their social anxiety disorder diagnoses post-training, and only 2 participants no longer met criteria for social anxiety disorder in the NOCBM group, which did not reflect a significant difference between groups (χ^2^(1) = 2.23, *p* = 0.14).

Associations between change in social anxiety (child and parent report and clinician severity ratings) and change in benign or negative interpretations were not statistically significant after correcting for multiple tests (Bonferroni-corrected significance criterion level α = 0.008) (see Table 4). Unsurprisingly, given the lack of significant associations, there was no evidence of indirect effects (using the PROCESS macro; Hayes, 2013) of CBM on social anxiety symptoms or severity via change in benign or negative interpretation.

## 4 Discussion

The current study is the first to investigate CBM-I in children with clinical levels of social anxiety. We administered an established method of modifying interpretation among children (with some adaptations aimed to standardise and enhance the procedure), however children with social anxiety disorder did not report significantly greater changes in benign or negative interpretation after receiving CBM-I training than those who did not (although differences in change in benign interpretations approached significance). While conclusions must necessarily be tempered by the lack of successfully training interpretation bias, changes in interpretation were not significantly associated with changes in social anxiety symptoms, severity or diagnoses. It is worth noting that there was a trend towards significance, with a medium effect size, for a change in clinical severity ratings, however, this was not in the direction expected. Specifically the mean scores indicated that clinical severity increased in the CBM group and decreased in the NOCBM group.

These results differ from those of Vassilopoulos et al. (2009) who found a significant reduction in symptoms of anxiety following CBM-I among (non-clinical) children with elevated social anxiety symptoms. Notably, however, in that study children reported significant reductions in negative interpretation following the CBM-I procedure. It is possible that with a clinical population a greater intensity of training is required to bring about change in interpretation. For example, in the first trial of multisession CBM-I with adults diagnosed with social anxiety disorder the CBM-I training used a word sentence association paradigm, and the intensity of CBM-I training was substantially higher (12 × 20 min over 6 weeks, with 220 training trials in each session) than in the present study, and this training was associated with both a reduction in negative interpretation and an increase in positive interpretation of novel social situations (Amir & Taylor, 2012). Following Vassilopoulos et al. (2009), there was a low dose of training in the present study, with only 45 training trials in total distributed over three sessions (i.e. 15 items per session). In comparison with most CBM-I multisession studies, this is a low dose both in terms of number of session and number of overall trials. The reason that we employed this method was based on its previous success and because we were concerned that children would not engage with CBM-I if too many sessions or trials were employed. However, it is notable that children of 10 years of age have been found to comply with large numbers of (albeit far briefer) trials in Attention Bias Modification (ABM) procedures (768 trials per session; Bar-Haim, Morag & Glickman, 2011) so more extensive CBM-I training with children may well be feasible. Notably the CBM-I appeared to be an acceptable intervention for children referred for treatment for social anxiety disorder and their parents. Consistent with adult studies in which participants are paid to take part (Beard et al., 2011), retention to this study (where there was no participant payment) was high, with only two of the 29 CBM-I participants failing to complete the training. No adverse effects of the training were reported. This suggests that future studies with greater therapeutic dose or where CBM-I is used as an adjunct alongside, for example, CBT (e.g. Beard et al., 2011; although see Williams et al., 2015) may be feasible interventions for children with social anxiety disorder.

It is important to note that in the current study, both the CBM-I and control groups experienced an increase in benign interpretation and a reduction in negative interpretation over time. It is unclear whether this is an artefact of the test used, regression towards the mean, or a non-specific effect of being part of a study that investigates social anxiety where an assessor meets with the child and parent. Furthermore, it has not been formally established that the two sets of the ambiguous scenarios measures of interpretation are equivalent, however Vassilopoulos et al. (2009) found no significant differences on scores on the two sets administered before and after a no-training condition. Clearly, before firm conclusions regarding the role of interpretation bias in maintaining clinical levels of social anxiety in children, or the potential utility of CBM-I in this population can be determined, effects of higher training dose needs to be trialed and more effective training methods need to be developed, including the use of CBM training and assessment materials that are specifically tailored for individuals with social anxiety disorder. For example a key concern in the context of social anxiety disorder is how the individual comes across to others, so materials which target this specifically may facilitate more effective training (e.g. Murphy et al., 2007).

The CBM-I training used here had previously been associated with a reduction in social anxiety following training among a high trait social anxiety community population (Vassilopoulos et al., 2009), however, in addition to the low dose, there are a number of reasons why the training may not have been optimal for application with clinically anxious children. Specifically, the CBM-I programme used here did not require participants to actively generate the meaning within trials, a factor which has been suggested to augment training effects (Mathews & Mackintosh, 2000). CBM-I studies have typically either involved active generation (Hoppitt, Mathews, Yiend & Mackintosh, 2010) or the use of imagery, with imagery having similar beneficial effects to active generation on mood in adults (e.g. Holmes & Mathews, 2010). Consequently, we adapted Vassilopoulos et al. (2009) methods to include generation of self-imagery. However, on reflection requiring children to generate images of themselves in the social scenarios may have been counterproductive. Indeed, Vassilopoulos, Blackwell, Moberly and Karahaliou (2012) recently found that children who read verbal descriptions and thought about their meaning showed a greater reduction in negative interpretation than children who imagined the events. Furthermore, social anxiety is associated with the generation of stereotyped negative self-images in socially anxious individuals (e.g. Hackmann, Clark, & McManus, 2000); it is therefore feasible that *negative* images were generated, that the children were unable to imagine themselves in the positive scenario, or even if they did so, that they did not believe that this is the way that the situation would actually go for them. Mathews, Ridgeway, Cook and Yiend (2007) used graded training where interpretation were initially benign and gradually moved on to more positive interpretation over time, minimising the potential for the training materials to be rejected. This may be a useful approach in future research.

Other considerations in interpreting inconsistencies in findings in relation to previous studies include differences in the method of administration of CBM-I in the current study which presented training materials via computer rather than experimenter (reducing the potential for experimenter bias). We also made other adaptations which were intended to augment training effects, however it is possible that these may have had the opposite effect. For example, we only presented benign comprehension questions at the end of each training item and did not provide feedback on responses to this. Furthermore, we made the aims of training explicit, but recent research by Grafton, Mackintosh, Vujic and MacLeod (2014) found that explicit instructions given during CBM, designed to facilitate positive attentional bias, led to poorer outcomes under stress conditions, potentially in keeping with the lack of transfer to symptoms of social anxiety in the current study (though see Krebs, Hirsch, & Mathews, 2010, for contrasting results highlighting the potential influence of the manner in which aims are made explicit).

Finally, the lack of a significant association between change in interpretation and change in social anxiety may reflect the possibility that interpretation biases do not have an independent causal role in relation to social anxiety in children, although the failure to successfully train a change in bias means we must be extremely cautious in drawing conclusions. However this suggestion would be consistent with recent findings that have failed to establish a tendency towards greater threat interpretation of social scenarios among children with social anxiety disorder (Creswell et al., 2013). Notably, however, these studies have typically failed to make a distinction between negative and benign interpretations. Future studies would benefit from consideration of developmental differences in the nature of the association between interpretation and social anxiety from childhood to adulthood.

Strengths of the current study were the inclusion of age and gender balanced groups from a referred population who all met diagnostic criteria for social anxiety disorder and consideration of potential confounding effects (behavioural disturbance and low mood). However, it is important to note certain limitations including the sample demographics (mostly high SES, Caucasian families) which limit the extent to which the findings can be generalised. We did not include a training control group, but as there were no differences in anxiety symptoms between the CBM and no intervention, this presents less of a problem in terms of interpretation. We also included children who met criteria for social anxiety disorder but this was not required to be their primary diagnosis. This may have meant that other interpretation biases were at play which may have accounted for the lack of translation of the effects of training to change in symptoms.

## 5 Conclusion

CBM-I training, adapted from methods successfully used with community populations, was not associated with significant changes in benign or negative interpretation in response to ambiguous information or changes in self, parent or clinician-reported social anxiety post-training. As such this study is not able to provide evidence relating to the causal influence of interpretation on social anxiety in children. Higher doses of CBM-I training are likely to be required to fully test this hypothesis.

## Figures and Tables

**Table 1:** Sample Characteristics at Baseline Assessment.

	CBM *N* = 29	NO CBM *N* = 27	
Age (months) mean (*SD*)	118.63 (17.11)	115.78 (19.29)	*t*(54) = −0.59, *p* = 0.56
Gender % female	66%	48%	χ^2^(1) = 1.72, *p* = 0.19
Family SES % ‘Higher professional’	59%	70%	χ^2^(1) = 1.61, *p* = 0.21
Ethnicity % White British	83%	89%	χ^2^(1) = 0.59, *p *= 0.44
SCAS-c Total mean (*SD*)	41.41 (16.95)	37.70 (12.99)	*t*(54) *=* −0.91, *p* = 0.37
SCAS-p Total mean (*SD*)	45.24 (11.23)	40.67 (15.55)	*t*(54) = −1.27, *p* = 0.21
SCAS-c Social phobia mean (*SD*)	7.55 (4.18)	6.30 (3.10)	*t*(54) = −1.27, *p* = 0.21
SCAS-p Social phobia mean (*SD*)	10.59 (4.10)	9.52 (4.58)	*t*(54) = −0.92, *p* = 0.36
SMFQ-c mean (*SD*)	6.62 (4.94)	5.78 (3.68)	*t*[54] = −0.72, *p* = 0.48
SMFQ-p mean (*SD*)	7.80 (5.83)	8.70 (4.59)	*t*(41) = 0.56, *p* = 0.58

*Note.* CBM: children who took part in training; NO CBM: children who did not take part in training; SD: standard deviation; SES: socio-economic status; SCAS-c: Spence Child Anxiety Scale – child version; SCAS-p: Spence Child Anxiety Scale – parent version; SMFQ-c: Short Mood and Feelings Questionnaire – child version; SMFQ-p: Short Mood and Feelings Questionnaire – parent version.

**Table 2:** Child Diagnostic Characteristics at Baseline Assessment.

	CBM	NO CBM
Diagnoses Primary (Overall) %	*N* *=* *29*	*N* *=* *27*
Separation Anxiety Disorder	24.1 (82.8)	29.6 (66.7)
Social Anxiety Disorder	31.0 (100)	25.9 (100)
Specific Phobia	10.2 (51.7)	0 (33.3)
Panic Disorder w/o Agoraphobia	0 (3.4)	0 (0)
Agoraphobia w/o Panic Disorder	0 (6.9)	0 (0)
Generalised Anxiety Disorder	31 (72.4)	44.4 (74.1)
Obsessive Compulsive Disorder	0 (0)	0 (3.7)
Selective Mutism	0 (0)	0 (7.4)
Anxiety Disorder NOS	3.4 (3.4)	0 (3.7)
Mood Disorder	0 (20.7)	0 (11.1)
Behavioral Disorder	0 (55.2)	0 (37.0)

*Note.* CBM: children who took part in training; NO CBM: children who did not take part in training; w/o: without; NOS: not otherwise specified. Mood Disorder: Depression/Dysthymia; Behavioral Disorder: Oppositional Defiant Disorder, Conduct Disorder, Attention Deficit/Hyperactivity Disorder.

**Table 3:** Pre- and post-training scores on measures of interpretation bias and anxiety.

	CBM Pre-Training	Post-Training	NO CBM Pre- Training	Post-Training
Interpretation	*N* *=* *28*		*N* *=* *27*	
ASI Benign (mean, SD)	24.93 (4.95)	32.68 (4.74)	23.89 (5.16)	28.33 (6.76)
ASI Negative (mean, SD)	21.89 (8.20)	18.21 (6.74)	23.63 (7.41)	21.22 (6.75)

Anxiety Self-Report	*N* *=* *29*		*N* *=* *27*	
SCAS SP Parent (mean, SD)	10.59 (4.10)	10.45 (3.16)	9.52 (4.58)	8.89 (3.87)
SCAS SP Child (mean, SD)	7.55 (4.18)	6.79 (4.17)	6.30 (3.10)	5.07 (4.51)

Anxiety Clinician Report	*N* *=* *29*		*N* *=* *27*	
CSR SP (mean, SD)	4.76 (.69)	4.97 (1.09)	4.70 (.72)	4.30 (1.59)

*Note.* CBM: children who took part in training; NO CBM: children who did not take part in training; SD: standard deviation; ASI: ambiguous scenarios interview; SCAS: Spence Child Anxiety Scale; SP: Social Phobia; CSR: Clinical Severity Rating.

**Table 4:** Correlations between difference scores of pre- and post- measures of anxiety and interpretation.

Anxiety	Interpretation	
	Benign	Negative
SCAS- p (Social Phobia)	−0.15	0.10
SCAS-c (Social Phobia)	−0.28	0.11
CSR	−0.05	0.01

*Note.* CSR: Clinical Severity Rating; SCAS-c: Spence Child Anxiety Scale – child version; SCAS-p: Spence Child Anxiety Scale – parent version.

## Footnotes

1 As different assessors interviewed the mothers and child simultaneously, reliability figures for parent and child report were calculated separately.
